# The impact of an additional extra-hepatic primary malignancy on the outcomes of patients with hepatocellular carcinoma

**DOI:** 10.1371/journal.pone.0184878

**Published:** 2017-09-28

**Authors:** Ming-Lun Yeh, Ching-I Huang, Chung-Feng Huang, Ming-Yen Hsieh, Zu-Yau Lin, Jee-Fu Huang, Chia-Yen Dai, Ming-Lung Yu, Shinn-Cherng Chen, Wan-Long Chuang

**Affiliations:** 1 Hepatitis Center and Hepatobiliary Division, Department of Internal Medicine, Kaohsiung Medical University Hospital, Kaohsiung, Taiwan; 2 School of Medicine, College of Medicine, Kaohsiung Medical University, Kaohsiung, Taiwan; 3 Health Management Center, Kaohsiung Medical University Hospital, Kaohsiung, Taiwan; 4 Cancer Center, Kaohsiung Medical University Hospital, Kaohsiung, Taiwan; National Yang-Ming University, TAIWAN

## Abstract

**Background:**

The impact of additional extra-hepatic primary cancer (EHPC) on the outcomes of patients with hepatocellular carcinoma (HCC) remains uncertain.

**Methods:**

We retrospectively analyzed the cancer registration database from a tertiary hospital in Southern Taiwan. Patients who were diagnosed with HCC from 2008 to 2012 were enrolled. Overall survival (OS), HCC-specific survival and recurrence after curative therapy were analyzed and compared between the patients with and the patients without EHPC.

**Results:**

EHPC was found in 121/1506 (8.0%) patients. HCC patients with EHPC were older, more likely to be classified as Child-Pugh A, less likely to have viral hepatitis B or C, more likely to be single, had early stage HCC and received curative therapy for HCC. The OS did not significantly differ between the patients with and without EHPC(p = 0.061). However, significantly higher HCC-specific survival was observed in patients with EHPC (p<0.001), and a higher rate of non-HCC mortality was demonstrated in patients with EHPC (54.4% vs 9.3%). The subgroup analysis revealed better OS in patients with EHPC who were older than 65, had viral hepatitis B or C, had non-stage 1 HCC, had non-early stage BCLC and received non-curative therapy. Conversely, patients with HCC stage 1 who received curative therapy exhibited worse OS if they also had EHPC. The analysis of recurrence after curative therapy showed no difference between the two groups.

**Conclusions:**

Our results implied that EHPC did not affect OS, but HCC-related survival was better in patients with EHPC. Based on these findings, the management of additional primary cancer is warranted.

## Introduction

Hepatocellular carcinoma (HCC) is the third leading cause of cancer death worldwide, with an estimated 598,000 deaths every year[1,2,3]. Several treatment strategies, including surgery, local ablation therapy to cure HCC, trans-arterial chemoembolization, and target therapy to control unresectable HCC, can be used in patients with HCC[4]. Combination treatment has also been studied in the setting of HCC but has only exhibited limited effects[5]. Disease status, including tumor stage, size, number and vascular invasion, was associated with the survival of patients with HCC[6,7]. Moreover, liver reserve function, socioeconomic status, and co-morbidity have also been shown to play important roles[8,9,10,11,12].

Improvements in anti-cancer treatments have prolonged the survival of individuals diagnosed with cancer, but this prolonged survival has also led to an increased risk of secondary primary malignancy. Specifically, concurrent extra-hepatic primary cancer (EHPC) represents a growing clinical challenge and may also influence the prognosis of patients with HCC[13]. Initial reports from a Japanese cohort demonstrated that a concurrent additional primary malignancy was not rare in patients with HCC and that the prognoses of these patients did not differ from those of patients without EHPC after HCC resection[14]. Another study in a Western cohort also demonstrated that males and older patients were more likely to harbor an EHPC[15]. However, survival did not differ between patients with EHPC and patients without EHPC.

Because of the significant variation in the distribution of chronic viral hepatitis and its association with HCC, the mechanism of carcinogenesis and the clinical presentation of patients with HCC differ by region[16]. To the best of our knowledge, the currently reported studies of HCC and EHPC were conducted in countries and regions with a low to intermediate incidence of HCC, and the presentation of HCC with EHPC in an area where HCC is highly prevalence as never been reported.

In the present study, we aimed to investigate the occurrence of EHPC in a large cohort of Taiwanese patients with HCC and clarify its clinical features, impact on patient survival and HCC recurrence.

## Patients and methods

The present study was a retrospective analysis of a cancer registration database at a tertiary hospital in Southern Taiwan. All patients with cancer must be registered in the database upon diagnosis at our hospital. We enrolled patients who were registered with HCC in the cancer registration database from January 2008 to December 2012. At our hospital, the diagnosis of HCC must meet at least one of the following criteria: 1) at least two radiological imaging assessments showing the typical features of HCC (early enhancement in the arterial phase and early wash-out in the portal venous phase), 2) one radiological imaging assessment showing the typical features of HCC associated with a serum alpha fetoprotein (AFP) level greater than 400 ng/mL, or 3) cytological/histological evidence. The diagnosis of HCC must have been confirmed by an HCC expert group for each patient. EHPC was diagnosed based on pathology and must have also been confirmed by our expert group of each primary cancer. Once each of the cancers was diagnosed, we must also register the cancer diagnosis and cancer stage to National Cancer Registration. Each patient’s geographic data, including gender, age, etiology, Child-Pugh score, AFP level, tumor number, tumor size, tumor TNM stage and Barcelona clinic liver cancer (BCLC) stage, were available from the database. The initial treatment, the tumor response (recurrence status after curative therapy) and the survival status could also be obtained from the database. In the present study, all the malignancy including HCC, EHPC received standard anti-tumor therapy according to the management guideline of our hospital.

The endpoints of the study included overall survival (OS, the period after HCC diagnosis until death or loss to follow-up), HCC-specific survival (the period after HCC diagnosis until death related to liver/HCC or loss to follow-up), and HCC recurrence (the period after curative therapy for HCC until the occurrence of recurrence). Once the death was confirmed in the hospital, the certification was provided and the death including causes of death was also reported to National Death Registration. The direct cause of death was ascertained by the ICD code of the direct cause of death on the death certificate. We determined HCC- or non-HCC-specific death by the ICD code. The recurrence of HCC was recorded in the database, which was confirmed by the clinical care physician. The final follow-up date for outcome assessment was December 31, 2014. For the patients who did not return to hospital in the scheduled period, we would get in touch the patients by telephone and confirm the status of patients. For the patients we could not get in touch, we set the patient as survival with the final date of visiting.

The study was approved by the Ethics Committee of Kaohsiung Medical University Hospital. (KMUHIRB-EXEMPT(II)-20160064) This study analyzed only aggregated secondary data without identifying specific patients, and the study protocol conformed to the ethical standards established by the 1964 Declaration of Helsinki, which waives the requirement for written or verbal patient consent in data linkage studies.

### Statistics

Continuous variables are expressed as the median, 25th percentile and 75th percentile, and the Mann-Whitney U test was used to compare continuous variables. Numbers and percentages were used to describe the distribution of categorical variables. Chi-squared and Fisher’s exact tests were used to compare categorical variables. Survival was analyzed with the Kaplan-Meier actuarial curve method with the log-rank test and the Cox regression hazard model. All tests were two-sided, and p <0.05 was considered significant. All analyses were performed using the SPSS 17.0statistical package (SPSS Inc., Chicago, IL, USA).

## Results

A total of 1506 patients who were diagnosed with HCC from 2008 to 2012 were enrolled. EHPC was found in 121 (8.0%) of the 1506 patients with HCC. Table 1 shows the clinical characteristics of all patients, with and without EHPC. The median age of all patients was 63.0 years, and nearly 70% of the patients were male. Seventy-three percent of the patients had compensated liver reserve function, and the primary etiology of HCC was viral hepatitis B or C. The median alpha fetoprotein (AFP) level was 46.6 ng/mL, and an AFP level ≥ 400 ng/mL was found in only 30.5% of the patients. Nearly 48% of the patients had HCC with only one tumor. The median size of the largest HCC was 4.0 centimeters (cm), and 38.5% of the patients had HCC tumors larger than 5 cm. Early stage HCC of TNM stage 1 and BCLC stage 0/A was noted in 39.7% and 40.0% of all patients, respectively. Five hundred three (33.4%) of the 1506 patients received curative therapy, including surgery and radiofrequency ablation (RFA), for HCC.

**Table 1:** Baseline characteristics of all patients with hepatocellular carcinoma (HCC) and the comparison between patients with/without extra-hepatic primary cancer (EHPC).

	All N = 1506	HCC with EHPCN = 121 (8.0)	HCC without EHPCN = 1385 (92.0)	p
Age, years	63.0 (55.0, 72.0)	67.0 (58.0, 74.0)	63.0 (55.0, 72.0)	0.011
Male gender	1045 (69.4)	81 (66.9)	964 (69.6)	0.539
Child-Pugh Classification A	1100 (73.0)	106 (87.6)	994 (71.8)	<0.001
Etiology of viral hepatitis B or C	1249 (82.9)	91 (75.2)	1158 (83.6)	0.023
Alpha fetoprotein, ng/mL*	46.6 (8.3, 933.5)	48.4 (8.4, 717.6)	46.5 (8.3, 999.3)	0.962
Alpha fetoprotein ≥400 ng/mL*	347 (30.5)	26/85 (30.6)	321/1051 (30.5)	1.000
Tumor number—Single	717 (47.6)	71 (58.7)	646 (46.6)	0.013
Tumor size (max), cm^†^	4.0 (2.4, 7.3)	3.2 (2.1, 6.3)	4.0 (2.4, 7.5)	0.025
Tumor size >5 cm^†^	527 (38.5)	34/112 (30.4)	493/1258 (39.2)	0.069
TNM stage 1	598 (39.7)	63 (52.1)	535 (38.6)	0.005
BCLC early stage (0/A)	603 (40.0)	58 (47.9)	545 (39.4)	0.067
HCC therapy—curative	503 (33.4)	51 (42.1)	452 (32.6)	0.035

*missing data for 370 patients

^†^missing data for 136 patients

Compared to the patients without EHPC, the patients with EHPC were significantly older (67.0 vs 63.0 years old, p = 0.011), had a higher incidence of compensated liver reserve function (87.6% vs 71.8%, p <0.001), had a lower incidence of viral hepatitis B or C etiology (75.2% vs 83.6%, p = 0.023), had a higher incidence of single HCC (58.7% vs 46.6%, p = 0.013), had smaller tumors (3.2 vs 4.0 cm, p = 0.025), had a higher incidence of TNM stage 1 HCC (52.1% vs 38.6%, p = 0.005) and were more likely to have received curative therapy (42.1% vs 32.6%, p = 0.035).

Of the 121 patients with EHPC, 92 (76.0%) patients harbored pre-existing EHPC before HCC diagnosis, and the remaining 29 (24.0%) patients developed EHPC after the diagnosis of HCC. Table 2 demonstrates the origins of the EHPC. The most common origin of EHPC was the digestive organs (32.2%), followed by the urinary tract and male genital organs (21.5%), and the lip, oral cavity and pharynx (19.8%). The other origins account for only one-fifth of EHPC.

**Table 2:** Sites of extra-hepatic primary cancer.

	N = 121
Digestive organs	39 (32.2)
Urinary tract and male genital organs	26 (21.5)
Lip, oral cavity and pharynx	24 (19.8)
Bone, skin and soft tissue	11 (9.1)
Breast	6 (5.0)
Lymphoid and hematopoietic	5 (4.1)
Unspecified sites	4 (3.3)
Respiratory and intrathoracic organs	3 (2.5)
Female genital organs	2 (1.7)
Thyroid	1 (0.8)

**Fig 1:**
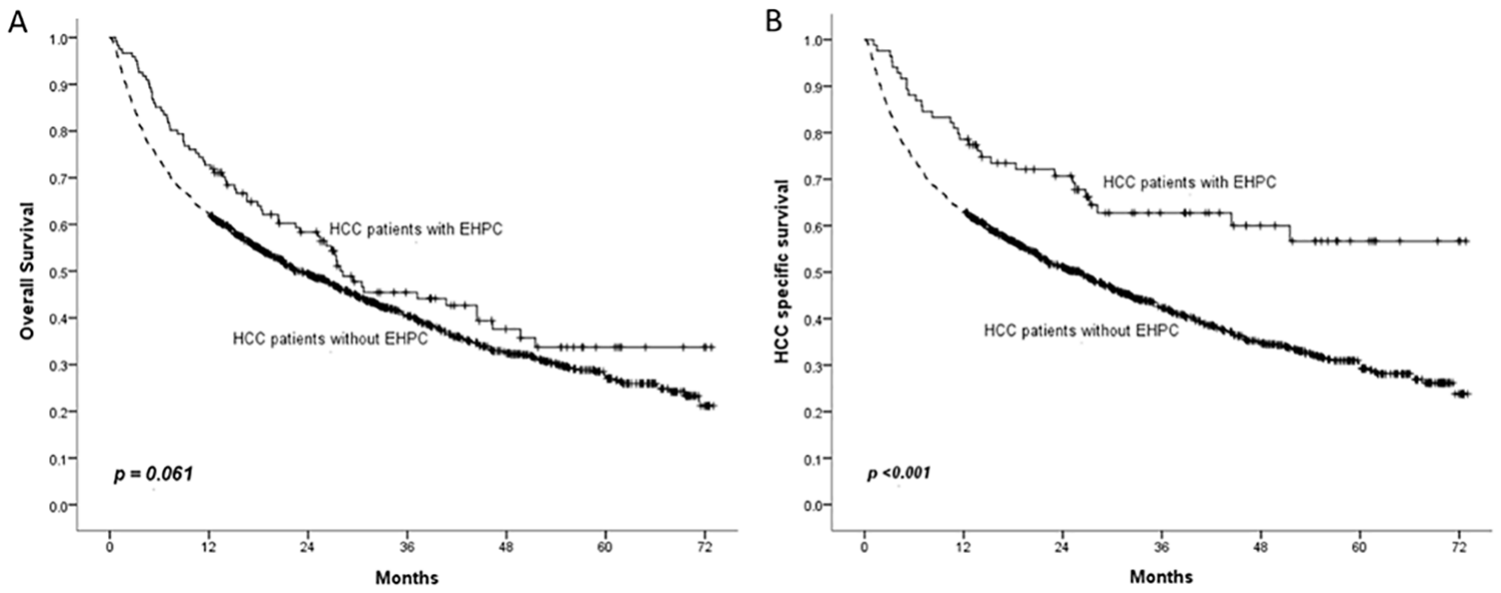
Comparison of overall and HCC-specific survival between patients with EHPC and patients without EHPC.

OS did not significantly differ between the patients with EHPC and the patients without EHPC (p = 0.061) (Fig 1A), but the HCC-specific survival rate was significantly higher in patients with EHPC than in patients without EHPC (p <0.001) (Fig 1B).

**Fig 2:**
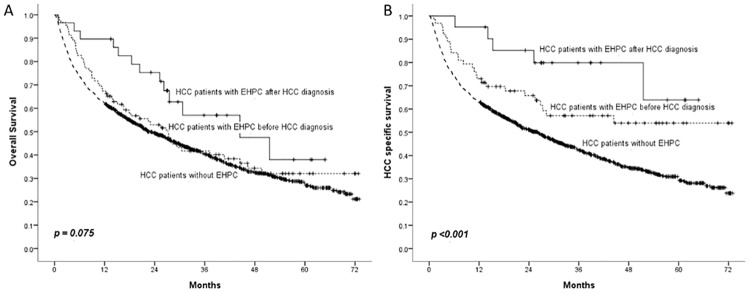
Comparison of overall and HCC-specific survival between patients with and patients without EHPC before and after HCC diagnosis.

Moreover, the data indicated that significantly more patients with EHPC died due to a non-liver/HCC etiology (37/68, 54.4%vs80/860, 9.3%, p <0.001). We further compared survival between the patients with EHPC before and after HCC diagnosis. The results revealed a significant difference in the OS between patients with EHPC and without EHPC after HCC diagnosis (p = 0.041). However, OS did not differ before and after HCC diagnosis among patients with EHPC (p = 0.117) or between patients with EHPC and without EHPC before HCC diagnosis EHPC (p = 0.331) (Fig 2A). The analysis of HCC-specific survival demonstrated significant differences between the patients with EHPC and without EHPC before HCC diagnosis (p = 0.007) and after HCC diagnosis (p = 0.006). However, HCC-specific survival did not differ before and after HCC diagnosis among the patients with EHPC (p = 0.133) (Fig 2B).

We analyzed the factors associated with overall and HCC-specific survival using the Cox regression hazard model. The initial analysis revealed that female gender (HR: 1.2, 95% CI: 1.03–1.37, p = 0.017), Child-Pugh A (HR: 3.8, 95% CI: 3.34–4.36, p <0.001), AFP <400 ng/ml (HR: 3.2, 95% CI: 2.74–3.74, p <0.001), BCLC stage 0/A (HR: 4.7, 95% CI: 4.04–5.52, p <0.001), curative therapy (HR: 4.3, 95% CI: 3.59–5.07, p <0.001), and the presence of EHPC after HCC diagnosis (HR: 1.8, 95% CI: 1.02–3.06, p = 0.041) were factors associated with overall survival. The multivariate analysis adjusted the significant factors in the univariate analysis and with EHPC revealed that EHPC was not associated with overall survival, either with EHPC before (Adjusted Model 2) or after HCC diagnosis (Adjusted Model 3). (Table 3)

**Table 3:** The Cox regression hazard analysis of factors associated with overall survival.

	Crude		Adjust Model 1		Adjust Model 2		Adjust Model 3	
	HR (95% CI)	p	HR (95% CI)	p	HR (95% CI)	P	HR (95% CI)	p
Female gender	1.2 (1.03–1.37)	0.017	1.0 (0.81–1.14)	0.657	1.0 (0.81–1.13)	0.609	1.0 (0.81–1.14)	0.632
Age ≥65 years	0.9 (0.80–1.04)	0.176						
Child-Pugh A	3.8 (3.34–4.36)	<0.001	2.8 (2.38–3.30)	<0.001	2.8 (2.41–3.35)	<0.001	2.7 (2.27–3.17)	<0.001
Viral etiology	1.1 (0.95–1.33)	0.180						
AFP <400 ng/ml	3.2 (2.74–3.74)	<0.001	2.1 (1.76–2.43)	<0.001	2.0 (1.72–2.38)	<0.001	2.1 (1.81–2.52)	<0.001
BCLC stage 0/A	4.7 (4.04–5.52)	<0.001	2.8 (2.24–3.34)	<0.001	2.7 (2.18–3.33)	<0.001	2.9 (2.32–3.58)	<0.001
Curative therapy	4.3 (3.59–5.07)	<0.001	0.4 (0.33–0.51)	<0.001	0.4 (0.33–0.51)	<0.001	0.4 (0.31–0.48)	<0.001
With EHPC	1.3 (0.99–1.62)	0.062	0.9 (0.71–1.26)	0.696				
With EHPC before HCC	1.1 (0.87–1.50)	0.331			0.9 (0.65–1.24)	0.495		
With EHPC after HCC	1.8 (1.02–3.06)	0.041					1.1 (0.57–2.03)	0.818

For HCC-specific survival, the univariate analysis showed that female gender (HR: 1.2, 95% CI: 1.02–1.38, p = 0.028), Child-Pugh A (HR: 4.1, 95% CI: 3.55–4.72, p <0.001), AFP <400 ng/ml (HR: 3.4, 95% CI: 2.86–3.99, p <0.001), BCLC stage 0/A (HR: 5.2, 95% CI: 4.37–6.15, p <0.001), curative therapy (HR: 4.9, 95% CI: 4.04–5.94, p <0.001), and the presence of EHPC (HR: 2.0, 95% CI: 1.39–2.86, p <0.001), with EHPC before HCC diagnosis (HR: 1.7, 95% CI: 1.16–2.54, p = 0.007) or with EHPC after HCC diagnosis (HR: 3.4, 95% CI: 1.42–8.24, p = 0.006), were all associated factors. However, EHPC either before or after HCC diagnosis was no more associated with HCC-specific survival in the multivariate analysis. (Table 4)

**Table 4:** Cox regression hazard analysis of factors associated with HCC-specific survival.

	Crude		Adjust Model 1		Adjust Model 2		Adjust Model 3	
	HR (95% CI)	p	HR (95% CI)	p	HR (95% CI)	P	HR (95% CI)	p
Female gender	1.2 (1.02–1.38)	0.028	1.1 (0.88–1.27)	0.541	0.9 (0.78–1.12)	0.491	0.9 (0.78–1.13)	0.51
Age ≥65	0.9 (0.81–1.07)	0.316						
Child-Pugh A	4.1 (3.55–4.72)	<0.001	2.8 (2.34–3.32)	<0.001	2.8 (2.35–3.33)	<0.001	2.7 (2.26–3.21)	<0.001
Viral etiology	1.1 (0.91–1.31)	0.369						
AFP <400	3.4 (2.86–3.99)	<0.001	2.1 (1.76–2.48)	<0.001	2.1 (1.73–2.44)	<0.001	2.1 (1.76–2.50)	<0.001
BCLC stage 0/A	5.2 (4.37–6.15)	<0.001	3.0 (2.36–3.71)	<0.001	3.0 (2.35–3.71)	<0.001	3.0 (2.38–3.78)	<0.001
Curative therapy	4.9 (4.04–5.94)	<0.001	0.4 (0.29–0.47)	<0.001	0.4 (0.29–0.47)	<0.001	0.4 (0.29–0.46)	<0.001
With EHPC	2.0 (1.39–2.86)	<0.001	1.4 (0.93–2.11)	0.109				
With EHPC before HCC	1.7 (1.16–2.54)	0.007			1.3 (0.82–2.03)	0.264		
With EHPC after HCC	3.4 (1.42–8.24)	0.006					0.5 (0.20–1.43)	0.209

We further analyzed the impact of EHPC on OS and HCC-specific survival according to different parameters. Although OS did not differ among the entire patient population, OS was better among patients with HCC and EHPC than among patients without EHPC who were aged older than 65 years (p = 0.022), had an etiology of viral hepatitis (p = 0.015), were diagnosed with HCC stage greater than 1 (p = 0.007), were categorized as BCLC intermediate/advanced stage (p = 0.015) and received non-curative therapy (p = 0.006) (Table 5). In contrast, OS was worse among patients with HCC and EHPC than among patients without EHPC who were categorized as HCC stage 1 (p = 0.039) and who had received curative therapy (p = 0.024). HCC-specific survival was better for patients with HCC and EHPC than patients without EHPC when the patients were stratified by gender (male, p = 0.002 and female, p = 0.021), age (<65 years, p = 0.034 and ≥65 years, p = 0.001), Child-Pugh class A (p = 0.008), the etiology of viral hepatitis (p <0.001),HCC stage greater than 1 (p = 0.001), BCLC intermediate/advanced stage (p = 0.001) and the receipt of non-curative therapy (p <0.001). Interestingly, HCC-specific survival did not differ among patients who demonstrated worsening OS.

**Table 5:** Comparison of overall survival and HCC-specific survival between patients with EHPC and patients without EHPC according to different parameters.

		Patient no. (with/without EHPC)	Mean (SD) mo of OS (with/without EHPC)	P	Mean (SD) mo of HCC-specific survival (with/without EHPC)	p
Gender	Male	81/964	35.2 (3.3)/31.3 (1.0)	0.150	47.7 (4.3)/32.6 (1.0)	0.002
	Female	40/421	41.0 (4.8)/34.9 (1.5)	0.242	50.3 (5.4)/36.0 (1.5)	0.021
Age	<65	53/757	33.7 (3.8)/33.7 (1.1)	0.690	46.0 (5.1)/34.7 (1.2)	0.034
	≥65	68/628	39.4 (3.7)/30.7(1.2)	0.022	49.7 (4.3)/32.2 (1.3)	0.001
Child-Pugh Classification	A	106/994	39.5 (2.9)/40.4 (1.0)	0.773	53.0 (3.5)/41.8 (1.0)	0.008
	B/C	15/391	24.0 (6.9)/12.5 (0.9)	0.076	26.1 (8.4)/12.8 (1.0)	0.075
Etiology	Viral	91/1158	40.8 (3.2)/32.5 (0.9)	0.015	51.4 (3.7)/33.7 (0.9)	<0.001
	Non-viral	30/227	25.2 (3.7)/31.6 (2.1)	0.738	32.4 (5.8)/33.2 (2.2)	0.480
Alpha fetoprotein	<400	59/730	37.1 (3.6)/40.5 (1.2)	0.623	49.8 (4.6)/42.1 (1.2)	0.119
	≥400	26/321	20.3 (4.0)/16.0 (1.2)	0.165	24.3 (5.2)/16.5 (1.3)	0.082
TNM stage	1	63/535	43.2 (3.7)/50.3 (1.2)	0.039	58.0 (4.0)/52.2 (1.3)	0.249
	2/3/4	58/850	31.3 (3.9)/21.1 (0.9)	0.007	39.5 (5.0)/21.8 (0.9)	0.001
BCLC stage	0/A	58/545	48.6 (3.6)/52.2 (1.1)	0.254	60.1 (3.7)/53.7 (1.2)	0.193
	B/C/D	63/840	27.2 (3.6)/19.2 (0.9)	0.015	37.0 (5.1)/19.9 (0.9)	0.001
Curative therapy	Yes	51/452	44.7 (4.1)/54.0 (1.3)	0.024	60.6 (4.2)/55.7 (1.3)	0.303
	No	70/933	31.9 (3.5)/22.1 (0.8)	0.006	40.3 (4.6)/22.9 (0.9)	<0.001

**Fig 3:**
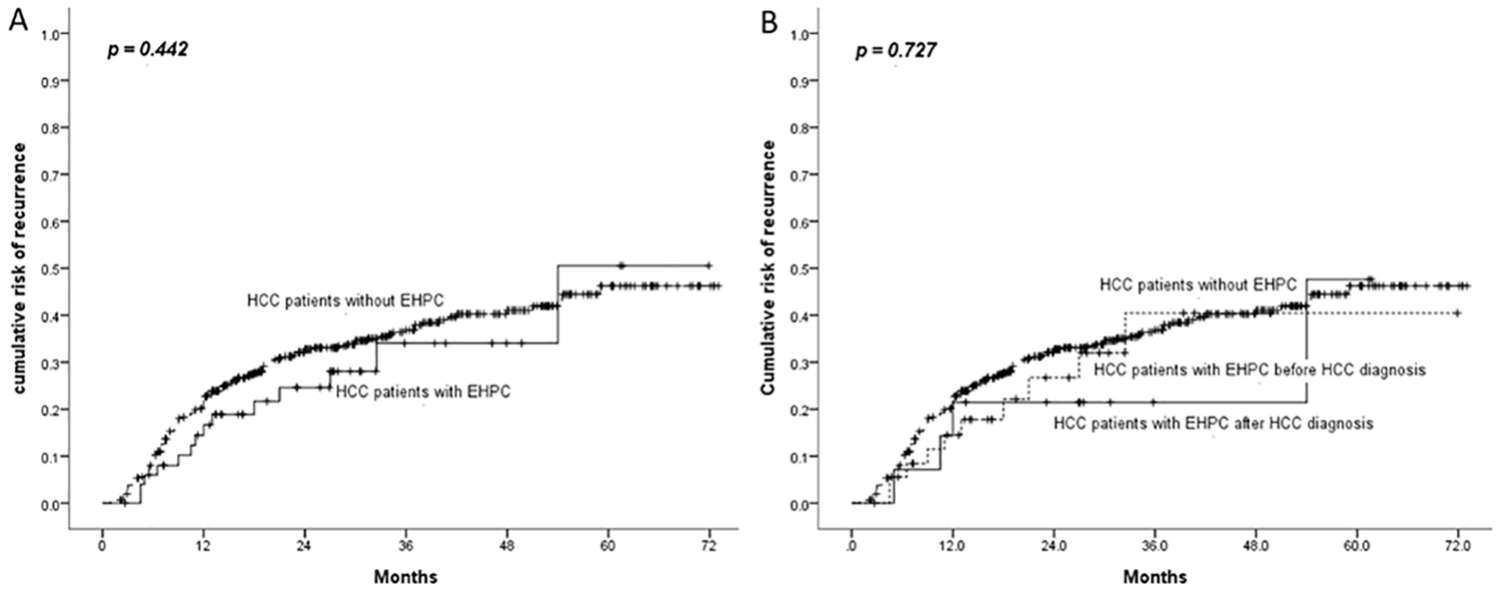
Comparison of HCC recurrence after curative therapy.

The analysis of the cumulative risk for HCC recurrence after curative therapy revealed no difference between the patients with and without EHPC (p = 0.442) (Fig 3A). Moreover, survival did not differ between the patients with EHPC and the patients without EHPC before or after HCC diagnosis (Fig 3B).

## Discussion

To the best of our knowledge, the present study examined the largest cohort in an area where HCC is highly endemic to investigate the impact of another primary cancer on patients with HCC. We found that a concurrent primary cancer was not uncommon and that most additional cancers developed before the diagnosis of HCC.

In the present study, we found that patients with EHPC were older, exhibited better liver reserve function, and were less likely to exhibit viral hepatitis etiology and harbored earlier-stage, smaller tumors. Moreover, American and Japanese cohorts of patients with EHPC were predominantly older and male[14,15]. Another study of a small Japanese cohort also demonstrated that viral hepatitis B and coexisting liver cirrhosis were less common among patients with EHPC[17]. However, a recent study of a Spanish cohort did not identify significant differences in the clinical characteristics or tumor status between patients with and without EHPC[18]. Our study is consistent with prior studies, showing that patients with EHPC are older and exhibit better liver function. However, our study is the first to describe a higher incidence of early stage HCC and smaller tumors among patients with EHPC. This finding was attributed to more frequent screening for liver metastasis in patients with EHPC.

As reported in previous studies, overall survival did not significantly differ between the patients with EHPC and without EHPC in our cohort of patients with HCC [18,19,20]. Interestingly, liver/HCC-related survival was significantly higher among the patients with EHPC in our cohort. This finding indicated that most patients with EHPC died from primary cancers other than HCC. This finding corroborated a recent report on a relatively small cohort from Korea[21]and was well-explained by the improved liver reserve function and the earlier HCC tumor stage among patients with EHPC compared with patients without EHPC. Moreover, the rate of recurrence after curative therapy, which had also never been studied, was also similar between the patients with and without EHPC. Moreover, the patients with EHPC who had an early HCC stage and received curative therapy showed worsening OS, but this difference disappeared when examining only HCC-related survival. This finding demonstrated that harboring another primary malignancy in addition to early stage HCC constituted a disadvantage.

In the present study, we did not find an impact of a second extra-hepatic malignancy on the survival of patients with HCC. With these results, we encouraged patients with HCC to maintain aggressive treatment for HCC even if a new second extra-hepatic malignancy is diagnosed. The results also encourage patients with HCC to acknowledge the possibility of a second extra-hepatic malignancy and to undergo surveillance for other malignancies, especially patients with related risk factors.

Nevertheless, the present study was also subject to limitations. First, the clinical parameters, such as viral load and antiviral therapy, which may have influenced survival, were not analyzed because these data were not available from the cancer registration database. Second, the details of the EHPCs, such as the stage and the anti-cancer treatment, were also unavailable.

In conclusion, the prevalence of EHPC in areas where HCC is highly endemic did not differ from that found in previous studies conducted in other regions. The presence of EHPC worsened overall survival or resulted in HCC recurrence. However, the patients with EHPC demonstrated a better HCC tumor status and better HCC-specific survival, especially older patients, patients with viral hepatitis-related HCC and patients with late-stage HCC. The results support the screening of patients with HCC for other primary malignancies and screening patients for other pre-existing cancers in areas where HCC is highly endemic. Furthermore, the results also imply the necessity of managing other primary cancers to decrease the rate of non-liver/HCC-related death.

## Supporting information

S1 Dataset Dataset of all the HCC and EHPC patients. (XLSX)
